# The complete chloroplast genome of balsam aster (*Aster ageratoides* Turcz. var*. scaberulus* (Miq.) Ling., Asteraceae)

**DOI:** 10.1080/23802359.2021.1955030

**Published:** 2021-07-27

**Authors:** Jie-Ying Feng, Yun-Zhe Wu, Rui-rui Wang, Xian-Fei Xiao, Rui-Hong Wang, Zhe-Chen Qi, Xiao-Ling Yan

**Affiliations:** a Zhejiang Province Key Laboratory of Plant Secondary Metabolism and Regulation, College of Life Sciences and Medicine, Zhejiang Sci-Tech University, Hangzhou, China; b Shaoxing Academy of Biomedicine of Zhejiang Sci Tech University, Shaoxing, China; c Eastern China Conservation Centre for Wild Endangered Plant Resources, Shanghai Chenshan Botanical Garden, Shanghai, China

**Keywords:** *Aster ageratoides*, Asteraceae, chloroplast genome, phylogenomic analysis

## Abstract

The first complete chloroplast genome of *Aster ageratoides* Turcz. var*. scaberulus* (Miq.) Ling. is reported in this study. The total chloroplast genome size of *A. ageratoides* var*. scaberulus* was 153,071 bp and comprised of a large single-copy region (LSC with 84,896 bp), a small single-copy region (SSC with 18,269 bp), and two inverted repeat regions (IR with 24,953 bp). A total of 122 genes were included in the genome, including 83 protein-coding genes, 8 rRNA genes, and 37 tRNA genes. Eleven protein-coding genes had intron (*ycf*3, *clpP* and *rps*12 gene contained two introns. Further phylogenomic analysis of Asteraceae, including 13 taxa, was conducted for the placement of *A. ageratoides* var*. scaberulus*.

*Aster ageratoides* Turcz. var*. scaberulus* (Miq.) Ling. (Asteraceae) is a perennial herb native to eastern Asia. It is widely distributed in China, Siberia Mongolia, South Korea and Japan (Chu et al. 2013). Because of its bright and daisy-like flowers, cultivars of *A. ageratoides* var*. scaberulus* are widely used as garden and cut flowers. Additionally, a recent study showed that aerial parts of *A. ageratoides* var*. scaberulus* are particularly rich in chlorogenic acid (CGA), which could potentially be used as a therapeutic agent in neurodegenerative disorders associated with glutamate excitotoxicity (Clifford et al. 2006; Rebai et al. 2017). Despite its importance in horticultural and medicinal value, there is little genetic information reported for *A. ageratoides* var*. scaberulus*. Here, we assembled and characterized the first complete chloroplast genome of *A. ageratoides* var*. scaberulus*. It will provide a valuable resource for further genetic conservation, evolution, and molecular breeding studies in the genus *Aster*.

Total DNA was extracted from dried leaves by DNA Plantzol Reagent (Invitrogen, Carlsbad, USA). The *A. ageratoides* var*. scaberulus* individual was collected from Chun’an, Zhejiang, China (GPS: E118°58′58.30″, N 29°46′56.71″). The specimen and extracted DNA were deposited at Zhejiang Province Key Laboratory of Plant Secondary Metabolism and Regulation, Zhejiang Sci-Tech University (http://sky.zstu.edu.cn) under the voucher number ZSTU00339 (collected by Zhe-Chen Qi, zqi@zstu.edu.cn). Whole plastome sequences were generated using the Illumina HiSeq-2500 platform (Illumina Inc., San Diego, CA, USA). In total, about 14.5 million high-quality clean reads (150 bp PE read length) were generated with adaptors trimmed. Aligning, assembly, and annotation was conducted by GetOrganelle (Jin et al. 2020) and GENEIOUS 11.1.5 (Biomatters Ltd, Auckland, New Zealand).

The full length of the *A. ageratoides* var*. scaberulus* chloroplast genome (GenBank Accession No. MW813970) was 153,071 bp and comprised of a large single-copy region (LSC with 84,896 bp), a small single-copy region (SSC with 18,269 bp), and two inverted repeat regions (IR with 24,953 bp). The overall GC content of the *A. ageratoides* var*. scaberulus* cp genome was 37.3% and the GC content of the LSC, SSC and IR regions are 35.2%, 31.3%, 43.1%. A total of 122 genes were included in the genome (83 protein-coding genes, eight rRNA genes, and 37 tRNA genes). Nineteen genes had two copies, which were comprised of seven PCG genes (*ndhB*, *rpl*2, *rpl*23, *rps*7, *rps*12, *ycf*1, *ycf*2), seven tRNA genes (*trnV-GAC*, *trnR-ACG*, *trnN-GUU*, trnL-*CAA*, *trnI-GAU*, *trnI-CAU*, *trnA-UGC*), and all five rRNA species (*rrn*16, *rrn*23, *rrn*4.5, *rrn*5, *rrb*16). In the genome, nine protein-coding genes (*rps*16, *rpo*C1, *atp*F, *pet*B, *pet*D, *rpl*16, *rpl*2, *ndh*B, *ndh*A) had one intron, and *ycf*3, *clp*P, *rps*12 gene contained two introns.

Twelve species with available chloroplast genomes were selected to study the phylogenetic placement of *A. ageratoides* var*. scaberulus* in Asteraceae. The alignment of plastomes was generated by MAFFT v7.475 (Katoh and Standley 2013). The maximum likelihood (ML) analysis was performed using IQTREE v 1.6.8 (Nguyen et al. 2015), of which the bootstrap values were calculated using 5000 replicates with the best-selected TVM + F+R2 model. The result showed that *A. ageratoides* var*. scaberulus* is sister to a clade formed by *A. tataricus*, *A. pekinensis*, *A. ataicus* and *A. flaccidus* according to the current sampling extent (Figure 1).

**Figure 1.**
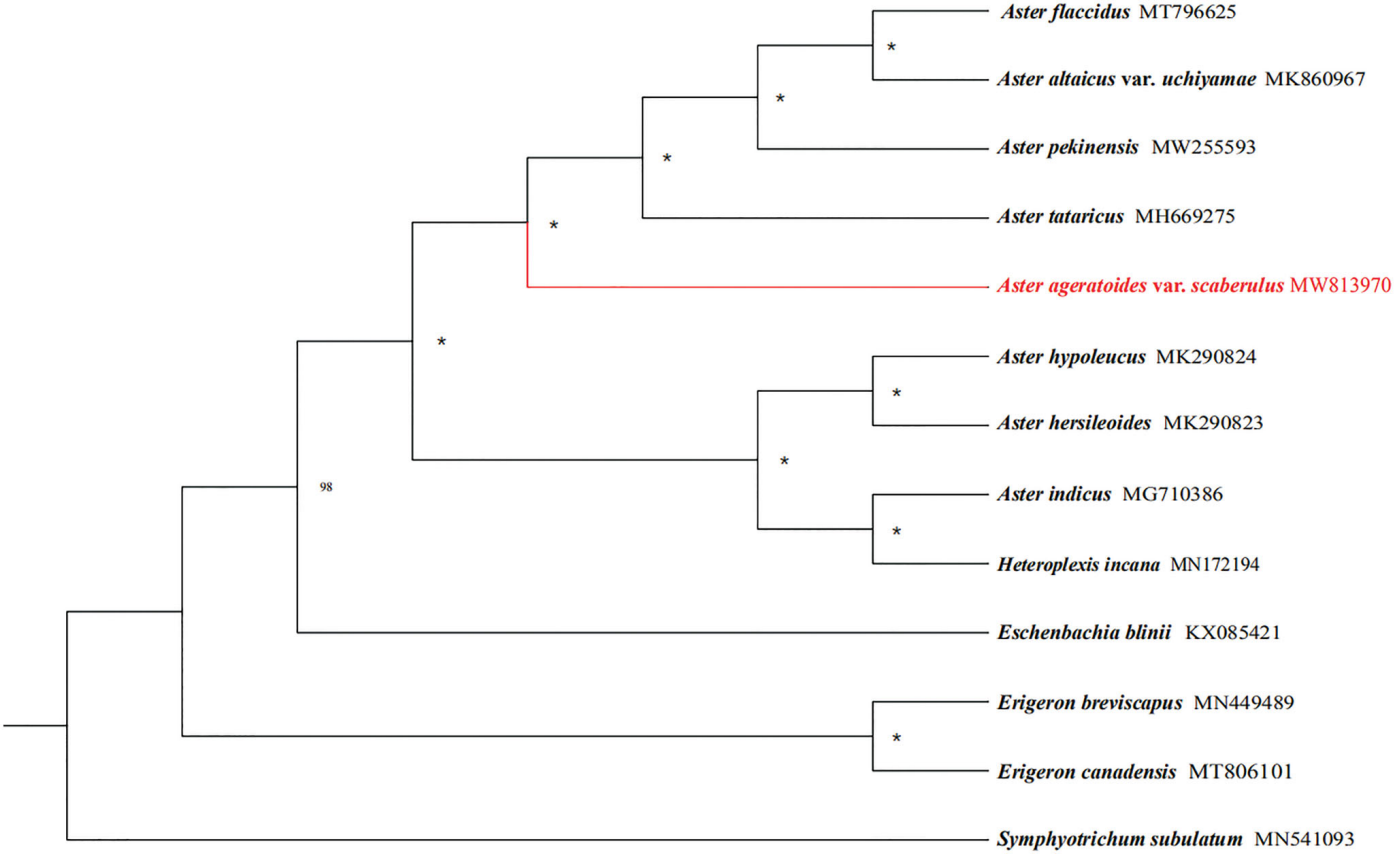
The phylogenetic tree based on 13 complete chloroplast genome sequences in Asteraceae (accession numbers were listed in front of their names and ‘*’ indicates the bootstrap support values = 100).

## Data Availability

The genome sequence data that support the findings of this study are openly available in GenBank of NCBI (https://www.ncbi.nlm.nih.gov) under the accession no. MW813970. The associated BioProject, SRA, and Bio-Sample numbers are PRJNA722057, SRR14241056, and SAMN18744330, respectively. The DNA matrix and phylogenetic tree that support the findings of this study are openly available in Github at https://github.com/andresqi/balsam-aster-plastome.

## References

[CIT0001] Chu SS, Liu LS, Liu ZQ, Jiang HG, Liu LZ. 2013. Chemical composition and insecticidal activities of the essential oil of *Aster ageratoides* flowering aerial parts. J Serb Chem Soc. 78(2):209–216.

[CIT0002] Clifford MN, Zheng W, Kuhnert N. 2006. Profiling the chlorogenic acids of aster by HPLC-MS(n). Phytochem Anal. 17(6):384–393. 17144245 10.1002/pca.935

[CIT0003] Jin J-J, Yu W-B, Yang J-B, Song Y, dePamphilis CW, Yi T-S, Li D-Z. 2020. GetOrganelle: a fast and versatile toolkit for accurate de novo assembly of organelle genomes. Genome Biol. 21(1):241. 32912315 10.1186/s13059-020-02154-5 PMC7488116

[CIT0004] Katoh K, Standley DM. 2013. MAFFT multiple sequence alignment software version 7: improvements in performance and usability. Mol Biol Evol. 30(4):772–780. 23329690 10.1093/molbev/mst010 PMC3603318

[CIT0005] Nguyen LT, Schmidt HA, von Haeseler A, Minh BQ. 2015. IQ-TREE: a fast and effective stochastic algorithm for estimating maximum-likelihood phylogenies. Mol Biol Evol. 32(1):268–274. 25371430 10.1093/molbev/msu300 PMC4271533

[CIT0006] Rebai O, Belkhir M, Sanchez-Gomez MV, Matute C, Fattouch S, Amri M. 2017. Differential molecular targets for neuroprotective effect of chlorogenic acid and its related compounds against glutamate induced excitotoxicity and oxidative stress in rat cortical neurons. Neurochem Res. 42(12):3559–3572. 28948515 10.1007/s11064-017-2403-9

